# Authenticity and Subjective Wellbeing within the Context of a Religious Organization

**DOI:** 10.3389/fpsyg.2017.01228

**Published:** 2017-07-19

**Authors:** Antonio Ariza-Montes, Gabriele Giorgi, Antonio Leal-Rodríguez, Jesús Ramírez-Sobrino

**Affiliations:** ^1^Department of Management, Universidad Loyola Andalucía Córdoba, Spain; ^2^Department of Business Administration, Universidad Autónoma de Chile Santiago, Chile; ^3^Department of Psychology, Università degli Studi Europea di Roma Rome, Italy

**Keywords:** authenticity, subjective wellbeing, religious organizations, partial least squares

## Abstract

Although authenticity has a long history as a philosophical and psychological idea, this concept has received scarce attention in the business literature until very lately. Nevertheless, scholars belonging to a broad array of disciplines have pointed out the escalation in the individuals’ search for authenticity within developed societies. Hence, the purpose of this paper is to assess the link between authenticity and subjective wellbeing within the rarely explored context of faith-driven organizations, where the management of emotions attains a particular significance. Specifically, this study links authenticity with subjective wellbeing among the distinct groups that shape a large international Catholic organization. This study uses Partial Least Squares (PLS) to test our research model and hypotheses. This paper covers two noteworthy research gaps. On the one hand, it provides evidence of the relationship between authenticity and subjective wellbeing within the context of religious organizations. On the other hand, our results suggest that this relationship is not homogeneous among the distinct groups that shape the organization. Implications of the research are finally discussed.

## Introduction

Over the past decades, sociologists and economists have begun to pay increasing attention to the study of faith-driven organizations (Miller, 2002). Among the reasons underlying this phenomenon, we highlight that such institutions have become major actors within certain segments of the third sector that are critical to maintaining the welfare state (i.e., education, healthcare, and social work, among others). Despite the serious conceptual problem in the study of the third sector [as Sajardo and Chaves, 2006 pointed out, the literature encompasses a variety of denominations regarding this complex amalgam of organizations: third sector, voluntary sector, non-profit sector, philanthropic sector, charity sector, non-governmental organization (NGO) sector, independent sector, tax-exempt sector, and social economy], the size of the non-profit sector within the entire global economy continues to grow and is currently a meaningful part of the European economic and social context. To assimilate the relevance of the third sector, Ayensa (2011) highlights that non-profit sector provides other major aspects that are difficult to quantify: first, how the social dimension of the sector broadens as it incorporates the impact made by voluntary work; second, the sector normally offers employment opportunities to some groups that are traditionally disadvantaged labor-wise, such as women, youth, and the disabled; finally, many third sector organizations provide services locally, thus capitalizing on the social fabric closest to home. In some specific sectors, non-profit organizations not only perform the largest proportion of the volunteer work but also tend to represent a very significant share of the paid employment (Hill, 2016). Scientific literature suggests that the division between voluntary and paid work is determined by two factors: the professionalism of the job and the time available. For this circumstance, the most complex and responsible positions are assigned to staff employed in the organization, whereas other easier and more sporadic activities are performed by volunteers with a much less stable commitment to collaboration.

Although this topic is undeniably relevant, these organizations have been rarely assessed at the organizational design and human resources management levels due to a poorly understood sense of shame or modesty, among other reasons. Sometimes a notion exists that discussions of labor contracts, wages, social benefits, work schedules, and other issues constitute taboo subjects, matters that to some extent betray the principles and values that underpin this type of organizations (Ariza-Montes and Lucia-Casademunt, 2013). This is why many non-profit organizations, including religious institutions, have been accused of making poor investments in terms of organizational infrastructure; for instance, in information technology (IT) systems, management skills, and other areas (Lowell et al., 2001). Consistent with these authors’ findings, the number of non-profit organizations that have implemented managerial training programs is certainly very low. Moreover, these duties are frequently the responsibility of inexperienced people who lack the capabilities to lead and/or motivate others (Lowell et al., 2001). Probably for these reasons, some authors as Bacchiega and Borzaga (2003), consider that non-profit organizations are only able to incorporate those employees who are not mainly influenced by remuneration of a monetary nature. The inability of non-profit organizations to attract and retain brilliant employees because of their low level of external competitiveness is a grave risk, which may put in danger its long-term survival.

The research gap with regard to the management of the distinct groups that shape faith-driven organizations seems to be clear. There is not even a general accepted definition of such organizations in the scientific literature. According to Ferris (2005), religious organizations might present one or more of the following features: affiliation with a religious group, a mission statement with explicit reference to religious values, financial support from religious sources, and/or a governance structure in which the selection of members of the Board and/or staff is based on religious beliefs or in which decision-making processes are based on religious values. These organizations represent a diffuse and plural context in which religious and lay people coexist and work together. Furthermore, the latter group also involves a certain level of heterogeneity that ranges from “professionals” to employees with labor connections and little commitment to the institutional objectives to (unpaid) volunteers who do not have contracts and who have high levels of commitment, although in many cases these individuals exhibit low levels of professionalism (Ariza-Montes and Velasco, 2006). This diversity implies an increase in the organization’s complexity due to the multiplicity of agents acting within the organization, which indicates the need for more scientific assessment of this topic.

Under this framework, it should be noted that most of the people who are professionally linked to the third sector, and especially to religious organizations, may be driven by ideological motivations – service vocation, personal self-actualization, identification with a set of values, and others (Elson, 2006). A fundamental part of the commitment to an organization’s ideology involves an expectation that the organization’s members accept the peculiarities that shape this type of institution: few professional staff, whose compensation tends to be low relative to the market and independent of payments or contributions from customers; strong dependence on volunteer and part-time work; and the existence of a congregational structure that reduces the need for full-time professionals while providing a source of support and reliability for the services they deliver (Iannaccone, 1992). The way in which faith-driven organizations develop their non-profit activities also involves several unique and idiosyncratic features. The objective of these organizations lies more in how they conduct their work than in how much work they accomplish. This style defines the character of a religious institution, a character that in some cases is formalized through internal documents aimed at serving as a guide for employees; for instance, the letter of identity (OHSJD, 2000) of the Brothers Hospitallers of Saint John of God or the Jesuits’ characteristic pedagogical letters (Compańía de Jesús, 1986). In the absence of formalization, the transmission of charisma happens through informal procedures such as socialization, which may be strengthened by some type of structure or supported by consecrated members and the rest of the staff.

Besides, according to Roof (2015), there is evidence to sustain the existence of a clear connection between spirituality and employee engagement. The intensification of the attention devoted to spirituality and employee engagement is due to a convergence of the following ten cultural dynamics: (i) recent ethical concerns, (ii) contemporary enlightened leadership theories, (iii) reaction to increasing materialism, (iv) more humanistic organizational environments, (v) a spiritual awakening in the workplace, (vi) a search for personal values, (vii) the rejection of greed, (viii) a shift toward wholeness and empowerment, (ix) a quest for employee meaning and purpose, and (x) the focus on positive psychology, meaningfulness, and wellness (Roof, 2015).

Given these idiosyncratic peculiarities, understanding how the members of this type of organization feel constitute a basic pillar for its long-term survival. Specifically, this study links authenticity or authentic behavior with subjective wellbeing among the distinct groups that shape a major religious organization. Being authentic involves being yourself, i.e., acting in a manner consistent with your beliefs and personal experiences. A growing body of research highlights the importance of being authentic for human functioning (Boucher, 2011; Grandey et al., 2012). This work adopts as a framework the tridimensional model of a person proposed by Wood et al. (2008) that suggests people are shaped by self-alienation, an authentic life and external influence acceptance. Although interest in authenticity has always existed, only recently has its connection with human wellbeing been elucidated (Addison, 2008). According to the current research in positive psychology, authenticity is considered to be at the core of wellbeing, as it not only constitutes a prior component or requisite to achieving wellbeing but is itself the essence of wellbeing (Wood et al., 2008).

Hence, the purpose of this study is to assess the link between authenticity and subjective wellbeing within a rarely explored context, the one shaped by faith-driven organizations, in which the management of emotions attains a particular significance. To this aim, we focus on a large international Catholic organization with a strong presence in the south of Spain and the Canary Islands. The labor performed by this organization is centered in the education sector (pre-university education) and in the social work sector, which is mainly composed of residences for elder people, orphanages and social dining rooms. Distinct groups coexist within this institution that differ in terms of their type of linkage with the organization: consecrated members, managerial laypeople and non-managerial laypeople (hereafter religious, managers and employees). The results obtained identify the main differences with regard to the feelings of authenticity among the distinct members and the link between authenticity and the level of subjective wellbeing the members attain. Ultimately, this study seeks to cover the research gap that exists for the authenticity-subjective wellbeing tie within the context of religious organizations.

To this aim, this paper continues as follows: in Section “Theoretical Background and Research Hypotheses” we present a review of the most relevant literature and posit our research model and hypotheses. Section “Materials and Methods” describes the methodology we followed in this study. Section “Results” presents the main results obtained from the empirical assessment of the hypothesized links. In Section “Discussion,” we discuss the most critical empirical results. The paper ends with the main implications, limitations and suggestions for future lines of research.

## Theoretical Background and Research Hypotheses

### Authenticity

Liedtka (2008, p. 237) posits that “Scholars across a wide variety of disciplines have called attention to what they see as an intensifying search for authenticity on the part of individuals in developed societies.” According to Knoll et al. (2015), authenticity has a long history as a philosophical and psychological idea but a short one as an empirical research concept. In fact, the study of authenticity constitutes a relatively recent endeavor. The first author referring to authenticity is Rogers (1961) who considers it, from a humanistic perspective, an attitude that enables the complete functioning of human beings. Since then, a growing body of research has stressed the importance of being authentic (for instance, Grandey et al., 2012) to the extent that currently it can be considered a central topic of research in psychology and other related fields (Van den Bosch and Taris, 2014a).

Authenticity can be broadly understood as the ability of an individual to act according to his or her true feelings, beliefs and core values (Harter, 2002). In other words, it refers to the thoughts, emotions, needs, desires, preferences, and beliefs about oneself, which are translated into actions that are consistent with those experiences (de Carvalho et al., 2015). According to these authors, being oneself is important because it produces beneficial effects for the development of individuals and groups, thus contributing to generating healthier organizations and social environments. A lack of authenticity produces anxiety and psychopathologies among individuals due to the necessity to develop forced or anti-natural behaviors. Moreover, several studies posit that fostering authenticity generates positive effects among the employees, as they experience greater enjoyment from and find more meaning in their job (Ménard and Brunet, 2011; Reich et al., 2013). However, the scientific literature suffers from a lack of empirical research on the assessment of authenticity in the workplace due primarily to the confusion that exists regarding this concept; in addition, until recently there have been few reliable measures of this construct (Sheldon, 2004; Wood et al., 2008). Most of the existing measures consider authenticity more as a stable trait than as a state related to a specific context (Metin et al., 2016).

Taking as a point of reference Rogers’s (1961) model that is centered on the person, Wood et al. (2008) define the tridimensional structure of authenticity, which is currently the most widely accepted model in the scientific community. The tridimensional construct posited by Wood et al. (2008) points to congruence as the critical element of authenticity, which is understood by Barrett-Lennard (1998) as the consistency between the three levels of (a) a person’s primary experience, (b) their symbolized awareness, and (c) their outward behavior and communication. The three dimensions of authenticity in the model by Wood et al. (2008) are self-alienation, an authentic life and external influence acceptance. **Self-alienation** is understood as the extent to which an individual experiences a certain level of incongruence between his or her conscience and an actual experience. If applied to the workplace setting, this notion would involve the subjective experience of not knowing very well who one is while at work. An **authentic life** involves being faithful to oneself in the majority of circumstances and living and acting in accordance with one’s own values and beliefs. Last, **external influence acceptance** refers to the degree to which one accepts the influence of other people at the same time that one is obliged to comply with the expectations of others (Wood et al., 2008). The optimal level of authenticity may be reached through a combination of high levels of authentic life and low levels of self-alienation and external influence acceptance.

In practice, being completely authentic becomes utopic, since as social beings all individuals are influenced in a way or another by their surrounding environment, which might in turn reduce authentic life while increasing the individual’s level of self-alienation. Indeed, the environmental pressures –real or imaginary– that saturate the atmosphere of some of these organizations might determine the degree of authenticity exhibited by the members of religious organizations (Chickering et al., 2015). Some people might presume that if they reveal themselves exactly as they are, they would be at risk of being recognized as different and banned or penalized in one way or another. The array of penalties is wide, ranging from social condemnation to the erosion of the possibilities for promotion and development of a professional career within the organization to an “invitation” to abandon the institution (Mengers, 2014). Consequently, individuals’ wellbeing tends to be severely damaged when they are forced to exhibit behaviors that are dissonant with their innermost feelings and patterns of acting while in the workplace (or in any other context of their life) and that compel them to renounce who they are. In this vein, Sheldon et al. (1997) suggest that individuals experience their behaviors as an expression of their true being in different contexts. Thus, greater authenticity might be positively related with superior levels of wellbeing and health.

### Subjective Wellbeing

Subjective wellbeing is a multifaceted and complex construct investigated in different scientific fields. In the framework of the self-determination theory, the assessment of wellbeing has developed into two distinct theoretical perspectives: hedonic and eudemonic (Deci and Ryan, 2008). Whereas the first relates to happiness, the latter is linked to a person’s potential development. The hedonic view of wellbeing identifies subjective happiness with personal pleasure, enjoyment and comfort. Therefore, as maintained by Diener (1984), there exists a direct tie between the hedonic approach and subjective wellbeing that is explained through three basic components: life satisfaction, the presence of positive feelings and the absence of negative feelings (Diener et al., 2003). Moreover, the eudemonic vision of wellbeing differs from subjective wellbeing, as the first focuses on the subjective experience of personal growth, self-actualization and life meaning or purpose, rather than on broad happiness (Ryan and Deci, 2001). Nonetheless, there are several authors who note the overlap that exists between both streams of thought (for instance, Huta and Ryan, 2010) to the extent that they have proposed that wellbeing is a multidimensional concept that covers aspects from both streams.

Assuming that any study of the topic of wellbeing demands the integration of both the eudemonic and hedonic approaches, this study relies on the use of the integrative perspective proposed by Diener et al. (2010). These authors consider that subjective wellbeing is a tridimensional construct shaped by two dimensions linked with the hedonic perspective (life satisfaction and the presence/absence of positive and negative feelings) and a third dimension grounded in both approaches (flourishing). Flourishing may reflect the essential components of wellbeing as revealed in the most recent theories (Sumi, 2014). From this point of view, flourishing describes an individual’s subjective perception of central areas of human activity that range from positive relationships to feelings of competence and self-esteem, as well as life meaning and purpose (Diener et al., 2010). Melé and Cantón (2014) describe human flourishing as the pursuit and fulfillment of the most noble human talents. This comprises a subjective feeling of happiness or personal joyfulness experienced by virtuous people. In any case, it is a mental health state characterized by high levels of subjective psychological and social wellbeing and associated with a significant proportion of positive affectivity (Cortina and Berenzon, 2013).

### The Authenticity-Wellbeing Link

The tie between being oneself and wellbeing represents a rather young research topic (Mengers, 2014). In fact, several psychological streams consider authenticity the most critical aspect of wellbeing given that, as Wood et al. (2008) note, being authentic is not only a prior component or requisite of wellbeing but also constitutes its core or essence. This interest has been intensified by the advent of positive psychology, a branch of psychology devoted to the scientific assessment of wellbeing that complements classic psychology by defining, researching and promoting “human flourishing” with regard to the study of mental health (Addison, 2008).

Several studies highlight that highly engaged employees tend to be more happy and productive (Rich et al., 2010; Leroy et al., 2013). In this vein, Avolio and Gardner (2005) suggest that authenticity involves being aware of one’s self and hence, adapting and behaving oneself in accordance. In this vein, Leroy et al. (2013) posit that when employees behave with authenticity at the workplace, thus experiencing that they behave as they wish, they will be more autonomously motivated for work-related issues, being hence, more likely to show personal engagement in those activities (Meyer and Gagné, 2008). Additionally, following Ariza-Montes et al. (2015), individuals who are authentically committed to work tend to be people meaningfully engaged in their setting –in a broad sense– rather than determined by their working conditions.

The link between authenticity and wellbeing seems evident to the extent that, as suggested by Sheldon et al. (1997), wellbeing may depend on the degree to which individuals show themselves authentically under different circumstances and with different people. Authenticity generates wellbeing by providing individuals with a clear and concise sense of themselves. In contrast, the absence of authenticity provokes disorientation and dissatisfaction, since individuals might be forced to act against their innermost values and aspirations. For instance, a study by Goldman and Kernis (2002) finds a strong correlation between authenticity, self-esteem and subjective wellbeing. Likewise, Wood et al. (2008) link authenticity with the increase of subjective wellbeing and the reduction of stress levels. Similarly, Neff and Harter (2002) identify lower levels of self-esteem and more depressive symptoms among individuals who subordinate or renounce their own interests with the aim of avoiding conflict, thus accepting external influence and feeling less authentic. Moreover, López and Rice (2006) measured authentic life and external influence acceptance (although not the self-alienation factor) and found significant correlations with depression, self-esteem, anxiety, and life satisfaction. The relationship between authenticity and life satisfaction has also been addressed in a study by Boyraz et al. (2014), which was carried out with a sample of students in two different periods of time. Specifically, the main drawback of most of these studies lies in the fact that they are not carried out within workplace contexts and they frequently rely on the use of samples of students; therefore, the constraints around the possibility of being authentic might differ.

In the labor context, Ménard and Brunet (2011) interviewed more than 300 executives, observing that those who scored higher in authenticity presented higher levels of subjective wellbeing in the workplace, although this relationship was partially mediated by the meaning that the executives found in their jobs. In the Australian healthcare sector, a study by Grandey et al. (2012) reveals that the most authentic workers present lower levels of strain than less authentic workers, who presented in turn higher emotional erosion.

Furthermore, a study by Van den Bosch and Taris (2014a), carried out with a wide sample of nearly 700 German employees, notes that authenticity in the workplace context explains a substantial quantity of the variance when predicting wellbeing. Applying a hierarchical regression model, these authors conclude that self-alienation constitutes the most determinant dimension of the authenticity construct. Hence, these authors maintain that authenticity is primarily grounded in the consistency between individuals’ primary experience and their symbolized awareness, that is, self-alienation.

All of the above leads to the formulation of the first research hypothesis of this work.

**Hypothesis 1**. *Authenticity is positively related to subjective wellbeing.*

As far as we know, there are no empirical studies linking authenticity with subjective wellbeing within the specific context of religious organizations, which constitutes a research gap that this work attempts to cover. The assessment of authenticity within this particular context is important because consistency between the behaviors – ways of acting – and beliefs of an individual constitutes a delicate and treacherous topic, especially in contrast to other types of organizations in which personal beliefs exclusively belong to the individual’s private dimension. Moreover, in these organizations, two types of collaborators coexist: (i) religious members who have professed vows and assume the role of owners–employees and (ii) lay members who present heterogeneous levels of identification with the institution’s ideology. The latter might in turn occupy executive positions with high responsibility or perform the role of base employees with lower responsibility and jurisdiction.

Similar to any other organization that acts within a certain environment, the survival and growth of religious institutions depends upon access to the resources existing within the environment. Their resources are frequently of a physical or financial nature, yet capacity to attract and retain members also becomes critical, as does those members’ levels of effort and commitment (Miller, 2002). This circumstance acquires even more relevance within the current scenario, which is characterized by the scarcity of religious vocations, leading to greater involvement by laypeople in the management of religious congregations. At this point, a fundamental question arises: Are laypeople actually free to act consistently with their personal beliefs and experiences or is their conduct determined by what they think their institution expects from them? Mengers (2014) asserts that a possible consequence of an “excess of authenticity” is being distinguished from others and labeled as different, which could lead to social rejection and ostracism. Furthermore, it should be noted that religious groups tend to confer greater status on those individuals who possess a deeper knowledge about religious practices, those who comply with norms and those who show that they are aligned with the values and charisma of the institution. According to Mengers (2014), intense social interaction together with the processes of training and mentoring are the principal instruments employed by these institutions to identify potential candidates who may be able to occupy positions of leadership and responsibility. As posited by Sosik et al. (2011), many of these managers cope with considerable pressure due to the necessity to satisfy everyone. A natural adaptive response might be that lay managers within religious institutions adapt their conduct to what they think the institution expects from them, which could later result in a crisis of authenticity that might hinder subjective wellbeing.

Therefore, this study expects that the level of authenticity, as well as its relationship with subjective wellbeing, might be different among the distinct members or groups that shape faith-driven organizations: religious members, lay managers, and lay employees. In this sense, we propose the following research hypothesis:

**Hypothesis 2**. *The authenticity-subjective wellbeing link varies among the groups considered.*

**FIGURE 1:**
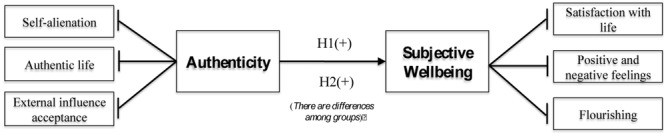
Research model and hypotheses.

The **Figure 1** illustrates the theoretical model and the research hypotheses.

## Materials and Methods

### Sample

To carry out this research, we prepared a survey that was sent to all the members of the target catholic organization (1942 in total) with several branches across Spain. The questionnaire was administered through Google Forms and was accompanied by a letter explaining the objectives of this study.

Google forms permits create and analyze surveys. This tool provides a fast way to create an online survey. After, the scholar can invite respondents by email. People answer questionnaire items from almost any web browser, including mobile smartphone and tablet browsers. Furthermore, survey responses gather in a spreadsheet saved to your Google Drive.

All subjects gave their informed consent for inclusion before they participated in the study. The study was conducted in accordance with the Declaration of Helsinki, and the protocol was approved by the Ethics Committee of Universidad Loyola Andalucía (Spain). The data collection was carried out between April and May 2016. Our two mailing efforts yielded a total of 1014 answered questionnaires; of those, we rejected 51 that contained some incomplete sections, resulting in a final sample of 963 valid questionnaires, which represents a 49.6% response rate. Of the respondents, 20.2% are religious, 6.1% are lay managers, and 72.7% are lay employees. In terms of sectors of activity, 55.2% work in the education sector (27.3% of the religious members, 86.7% of the lay managers, and 61% of the lay employees), and the remaining 44.8% work in the social assistance sector, which is composed primarily of residences for elderly people, homes for orphans and social dining rooms (72.7% of the religious members, 13.3% of the lay managers, and 39% of the lay employees).

The majority of the survey respondents are women (84.2% of the total sample, 91.4% of the religious members’ subsample, 73.3% of the lay managers’ subsample, and 83% of the lay employees’ subsample), with an average age of 44.9 years old (*SD* = 11.8) (51.9, 46.7, and 42.8, respectively) and an average seniority of 17.7 years (*SD* = 9.6) (21.8, 17.7, and 12.8, respectively). Of those surveyed, 69.4% reported that they had completed university studies, 19% had finished secondary studies, and 11.6% have primary education. Finally, 58.2% of those surveyed live with a partner, 22.9% live alone, and the remaining 18.9% live in community.

### Measures

The authenticity variable was measured using the IAM (*Individual Authenticity Measure at Work*) developed by Van den Bosch and Taris (2014a). This instrument, composed of 12 items, is an adaptation for the workplace context of the authenticity scale developed by Wood et al. (2008). Consistent with the original scale, the IAM comprises three dimensions: self-alienation (i.e., “At work, I feel out of touch with my true self”), authentic life (i.e., “At work, I am faithful to myself in most situations”), and external influences acceptance (i.e., “At work, I feel the need to do what others expect me to do”). All the items were measured using a Likert scale ranging from 1 (totally agree) to 5 (totally disagree). Later, we recoded the self-alienation and external influence acceptance scales. Therefore, consistent with the scale for an authentic life, a higher score represents a higher degree of authenticity. The scale’s reliability can be found in studies by Van den Bosch and Taris (2014a) and Metin et al. (2016). The reliability estimates in this study for the three dimensions ranged from 0.741 (authentic life) to 0.782 (external influences acceptance).

To measure subjective wellbeing, we relied on the use of a scale developed by Diener et al. (2010). This instrument encompasses three dimensions: (a) satisfaction with life (*Satisfaction With Life Scale-SWLS*), (b) positive and negative experiences (*Scale of Positive and Negative Experience-SPANE*), and (c) flourishing (*Flourishing Scale-FS*). The Cronbach’s alpha for this scale was 0.814.

(a) Satisfaction with life (SWLS) is measured through five items that assess the global judgment that people make concerning their level of satisfaction with their life. Example items from the scale include “I am satisfied with my life” and “Until now I have achieved all the important goals that I have set in life.” The respondents answered using a scale ranging from 1 (totally disagree) to 5 (totally agree). The psychometric properties of this scale are detailed in studies by Diener et al. (2010), Dogan and Totan (2013), or Moyano et al. (2013). The Cronbach’s alpha for SWLS scale was 0.874.
(b) The scale of positive and negative experiences (SPANE) consists of a questionnaire of 12 items including six positive feelings (feeling well, happy, kind, etc.) and six negative feelings (feeling bad, frightened, sad, etc.). The respondents indicated the extent to which they feel one way or another using a scale ranging from 1 (very rarely or never) to 5 (very often or always). Studies by Silva and Caetano (2013) or Sumi (2014) validate this measurement instrument. Our research obtained an alpha coefficient of 0.900 (positive feelings) and 0.793 (negative feelings).
(c) The flourishing scale (FS) used is an adaptation for the workplace context that Mendonça et al. (2014) developed from the original scale proposed by Diener et al. (2010). This instrument comprises eight items that measure the respondents’ perception of critical aspects of human functioning such as relationships, purpose in life or optimism. Example scale items include “My work contributes to having a meaningful life and purpose” or “At work, people respect me.” The respondents may indicate the extent to which they agree with the different statements using a scale ranging from 1 (totally disagree) to 5 (totally agree). The psychometric properties of this scale can be found in the study by Mendonça et al. (2014). In our study, the Cronbach’s alpha was 0.906.

### Data Analysis

The research model described in **Figure 1** has been tested through the use of partial least squares (PLS) path modeling, a variance-based structural equation modeling (SEM) technique (Roldán and Sánchez-Franco, 2012). PLS simultaneously enables the assessment of the reliability and validity of measures of theoretical constructs (outer model) and the estimation of the relationships among these constructs (inner model) (Barroso et al., 2010).

The PLS methodology is suitable for studies conducted within the social sciences research field for the following reasons: (i) the measurement scales are frequently poorly developed; (ii) the phenomena investigated are relatively new or in progress and theoretical frameworks lack solid development; (iii) the data tend to be non-normally distributed; (iv) there are sufficient ordinal and categorical data; (v) the focus is more on the prediction of the dependent variables than in the confirmation and fit of the model; and (vi) the research model happens to be very complex relative to the type of relationships stated in the hypotheses (Roldán and Sánchez-Franco, 2012). The PLS methodology is suitable for studies conducted within the social sciences research field for the following reasons: (i) the phenomena investigated are relatively new or in progress and theoretical frameworks lack solid development; (ii) the focus is more on the prediction of the dependent variables than in the confirmation and fit of the model; and (iii) this technique enables the use of component scores in a subsequent analysis for modeling multidimensional constructs, applying the two-stage approach (Chin, 2010; Roldán and Sánchez-Franco, 2012). We used the SmartPLS 3.0 software to statistically test the measurement and structural models (Ringle et al., 2015).

We followed a two-step approach to operationalize the multidimensional superordinate constructs (Chin, 2010). Accordingly, the items for each dimension were optimally weighted and combined using the PLS algorithm to create a latent variable score. Consequently, the dimensions or first-order factors became the observed indicators of the second-order constructs, which are the authenticity and subjective well-being variables (Chin and Gopal, 1995).

## Results

### Descriptive Statistics

With regard to the central variables in this study, **Table 1** presents the main descriptive statistics, the Cronbach alpha for each analyzed construct, and the bivariate correlations between the main research variables. As it can be observed, the surveyed show elevated levels of authenticity (3.97 on a maximum of 5). At the same time, these subjects denote a high level of subjective wellbeing in all its dimensions (flourishing: 4.44; satisfaction with life: 3.92; and positive–negative feelings balance: 2.18).

**Table 1:** Descriptive statistics, Cronbach’s alpha and inter-correlations for the study variables.

Variable	Mean	*SD*	Cronbach’s alpha	1	2	3	4
1. IAM	3.97	0.656	0.793	1			
2. SWLS	3.92	0.796	0.874	0.216^∗∗∗^	1		
3. SPANE	2.18	1.199	0.900	0.285^∗∗∗^	0.473^∗∗∗^	1	
4. FS	4.44	0.712	0.906	0.369^∗∗∗^	0.449^∗∗∗^	0.353^∗∗∗^	1

*^∗∗∗^*p* < 0.001.*

**Table 1** also reveals that the main variables are significantly related between each other, which is consistent with the most relevant research theories and hypotheses mentioned above.

### PLS Models

Partial least squares models are assessed in two stages: (i) verifying the reliability/validity of the measurement model and (ii) weighing the significance of the paths within the structural model.

#### Measurement Model

The assessment of the measurement model shows acceptable results. First, the indicators and dimensions satisfy the requirement of reliability because their loadings are, in general, greater than 0.707 (see **Table 2** for the general model with second-order constructs of AU and SW, and see **Table 3** for the same model but using the first-order dimensions of the AU construct). Only a few of the outer loadings are slightly below this critical level. Nevertheless, the decision was made to retain them to support the content validity of the scale.

**Table 2:** Measurement Model 1.

	Outer loadings			Construct reliability and validity			
	AU	SW		Cronbach’s alpha	rho_A (Dijkstra-Henseler’s indicator)	Composite reliability	AVE (average variance extracted)
EIA	0.629		AU	0.781	0.743	0.702	0.565
AL	0.853		SW	0.738	0.729	0.812	0.592
SA	0.607		**Discriminant validity**					
LS		0.813	***Fornell-Larcker***			***Heterotrait-Monotrait Ratio (HTMT)***		
PF		0.764		AU	SW		AU	SW
			
NF		–0.587	AU	*0.752*		AU		
FL		0.761	SW	0.511	0.769	SW	0.691	

*AU, authenticity; SW, subjective wellbeing; EIA, external influence acceptance; AL, authentic life; SA, self-alienation; LS, life satisfaction; PF, positive feelings; NF, negative feelings; FL, flourishing.*

**Table 3:** Measurement Model 2.

	Outer loadings					Construct reliability and validity			
	EIA	AL	SA	SW		Cronbach’s alpha	rho_A (Dijkstra-Henseler’s indicator)	Composite reliability	AVE (average variance extracted)
V1	0.666				EIA	0.777	0.789	0.899	0.817
V2	0.903				AL	0.783	0.788	0.874	0.699
V3	0.886				SA	0.835	0.87	0.901	0.752
V4	0.633				SW	0.772	0.735	0.81	0.589
V5		0.858				**Discriminant validity: *Fornell-Larcker***			
V6		0.543				EIA	AL	SA	SW
	
V7		0.838			EIA	0.904			
V8		0.771			AL	0.202	0.836		
V9			0.426		SA	0.445	0.241	0.867	
V10			0.803		SW	0.21	0.64	0.229	0.767
V11			0.905			**Discriminant validity: *Heterotrait-Monotrait Ratio (HTMT)***			
V12			0.893			EIA	AL	SA	SW
	
LS				0.809	EIA				
PF				0.764	AL	*0.258*			
NF				–0.519	SA	0.542	*0.295*		
FL				0.772	SW	0.301	0.814	*0.287*	

*AU, authenticity; SW, subjective wellbeing; EIA, external influence acceptance; AL, authentic life; SA, self-alienation; LS, life satisfaction; PF, positive feelings; NF, negative feelings; FL, flourishing.*

Second, all the second-order reflective (superordinate) and first order constructs meet the requirement of construct reliability because their composite reliabilities (CR), Cronbach’s alpha and Dijkstra-Henseler’s indicator (rho_A) are greater than 0.7.

Third, these latent variables attain convergent validity because their average variance extracted (AVE) surpasses the 0.5 critical level (**Tables 2, 3**). Lastly, **Tables 2, 3** reveal that all the variables achieve discriminant validity according to both the Fornell-Larcker and the HTMT criterion (Henseler et al., 2015). According to Fornell-Larcker criterion, diagonal elements (italics in **Table 2**) are the square root of the variance shared between the constructs and their measures (AVE). For discriminant validity, diagonal elements should be larger than off-diagonal elements. Off-diagonal elements are the correlations among the constructs. Finally, Heterotrait-Monotrait Ratio (HTMT) criterion should be under the threshold of 0.85 (Kline, 2015).

#### Structural Models

Consistent with Hair et al. (2011), a bootstrapping technique (5,000 re-samples) is employed to generate standard errors and t-statistics that permit the assessment of the statistical significance of the links considered within the two research models: Model 1 using second-order constructs for the total sample and Model 2 using the dimensions of authenticity, both for the total sample (Model 2A) and for each one of the distinct groups (Models 2B, 2C, and 2D). **Table 4** includes the main parameters obtained for the five models.

**Table 4:** Structural Models.

Relationship	Model 1 R^2^_SWB_ = 0.338		Model 2A *R*^2^_SWB_ = 0.382		Model 2B *R*^2^_SWB_ = 0.294		Model 2C *R*^2^_SWB_ = 0.406		Model 2D *R*^2^_SWB_ = 0.335	
	Path coefficient (t-statistic)	*p*-value	Path coefficient (t-statistic)	*p*-value	Path coefficient (t-statistic)	*p*-value	Path coefficient (t-statistic)	*p*-value	Path coefficient (t-statistic)	*p*-value
AU→SWB	0.582^∗∗∗^ (20.106) [0.530; 0.625]	0.000								
AEI→SWB			0.077^∗^ (1.728) [0.013; 0.132]	0.052	–0.128ns (0.774) [-0.323; 0.168]	0.168	0.051ns (1.335) [-0.022; 0.105]	0.091	–0.304ns (0.993) [-0.709; 0.063]	0.160
AL→SWB			0.577^∗∗∗^ (14.898) [0.507; 0.634]	0.000	0.499^∗∗∗^ (5.951) [0.345; 0.616]	0.616	0.583^∗∗∗^ (12.079) [0.492; 0.650]	0.000	0.488^∗∗∗^ (3.769) [0.220; 0.653]	0.000
SA→SWB			0.073^∗^ (2.196) [0.017; 0.127]	0.014	0.012ns (0.157) [-0.108; 0.145]	0.145	0.123^∗∗^ (2.917) [0.054; 0.194]	0.002	0.053ns (0.374) [-0.077; 0.4331]	0.354

*t-values in parentheses. Bootstrapping 95% confidence intervals bias corrected in square brackets (based on *n* = 5000 subsamples).*

*^∗∗∗^*p* < 0.001; ^∗∗^*p* < 0.01; ^∗^*p* < 0.05 [based on *t*(4999), one-tailed test]. *t*(0.05, 4999) = 1.645; *t*(0.01, 4999) = 2.327; *t*(0.001, 4999) = 3.092; ns, not significant.*

The main criterion that we use to evaluate the explained variance of the endogenous construct is the *R*^2^ coefficient. In this vein, the results presented in **Table 4** validate the different structural models assessed in this study, showing that they present acceptable predictive relevance for the dependent construct.

Model 1 describes the significant direct effect (path coefficient: 0.582^∗∗∗^; *t*-value: 20.106) of authenticity on subjective wellbeing for the entire sample (religious members, lay managers, and lay employees). This result leads us to conclude that there is empirical evidence to sustain our first hypothesis (H1).

Subsequently, in Model 2, we explore how each of the three dimensions of authenticity (external influence acceptance, authentic life and self-alienation) impact subjective wellbeing, and we assess these effects for the total sample – Model 2A – and for the three different subsamples that compose each of the categorized groups: Models 2B (religious members), 2C (lay employees), and 2D (lay managers).

As shown in **Table 4**, there are substantial differences with regard to the sign and significance of the three direct links represented within Models 2A–D. This fact contributes support for our second hypothesis (H2), which with exploratory character states that there are differences among groups with regard to the relationship between authenticity and subjective wellbeing. We explore and discuss such differences in depth in the discussion section.

## Discussion

The current study addresses this matter directly by focusing on the analysis of the relationship between authenticity and subjective wellbeing. Although interest in authenticity has existed for centuries, only recently has its link with wellbeing been empirically tested (Mengers, 2014). Individuals who feel more authentic and remain faithful to their own feelings and values exhibit more positive signs of wellbeing (Wood et al., 2008). This topic, which has been rarely addressed in general terms, presents an even more important research gap in the context of faith-driven organizations, as, to our knowledge, there are no studies that have framed the authenticity-wellbeing link within the religious institutions context. The absence of results from empirical analyses are certainly paradigmatic. On the one hand, these organizations have a significant presence within critical welfare sectors (i.e., social services, education, or healthcare). On the other hand, feeling comfortable and acting consistently with one’s own personal beliefs and experiences might be determinant of the development of feelings of belonging to the distinct groups that shape faith-driven organizations.

Given the above, this study has gathered information from nearly a thousand members of the education and social service sections of an international Catholic-inspired religious organization.

The notion underlying our first research hypothesis is that a positive link exists between feeling more authentic and subjective wellbeing. The PLS path-modeling used to test the hypotheses revealed that the more authentic members, those who reveal themselves as they truly are and how they truly feel without acting or behaving with hypocrisy, exhibit higher levels of subjective wellbeing and the construct shaped by satisfaction with life; a better balance between positive and negative experiences; a more positive self-perception of relationships; and more positive feelings regarding competence, self-esteem and purpose in life (flourishing). This result provides evidence to support Hypothesis 1. Thus, the first major contribution of this study lies in demonstrating that the tridimensional model for authenticity (concretely, the short version of the IAM) constitutes a significant predictor of subjective wellbeing in the workplace in the specific and rarely explored context of religious organizations. This evidence is consistent with the results found by other authors in different contexts such as that of bank employees (Metin et al., 2016), German employees within distinct sectors of activity (Van den Bosch and Taris, 2014b), managers of public organizations (Ménard and Brunet, 2011), and employees within the building industry in Singapore (Toor and Ofori, 2009).

The second main objective of this research was to assess whether the three components of authenticity – authentic life, external influence acceptance and self-alienation – exert a direct influence on an individual’s level of subjective wellbeing and, if they do, to explore whether this relationship is homogeneous among the distinct groups assessed or if there are differences among them.

Overall, Model 2A, as presented in the results section, indicates that the three components of authenticity determine the level of subjective wellbeing of the members of the religious institution under study, particularly with respect to remaining faithful to oneself (t-statistic: 14.898), although external influence acceptance (*t*-value: 1.728) and self-alienation (t-statistic: 2.196) are also significant. This result suggests that the core component of authenticity, the element that heavily influences subjective wellbeing, is authentic life, specifically, the extent to which the members of an organization are able to act consistently with their own values and personal beliefs. This component of the tridimensional model of authenticity is the strongest one within the general model and the one that determines with the greatest intensity the level of authenticity among the three groups under research: religious members, lay managers, and lay employees. Therefore, the probability of experiencing wellbeing in the workplace may be higher among those members who can act in an authentic way.

This tridimensional conceptualization of authenticity, applied in the context of a faith-driven organization, is consistent with the one developed by de Carvalho et al. (2015) in a study of a sample of approximately 500 Brazilian employees. In that study, the three elements of authenticity correlated with some of the measures of subjective wellbeing utilized by these authors: with flourishing in three cases and with satisfaction with life in all the cases except for external influence acceptance. Moreover, the strongest correlations were found for self-alienation, followed by authentic life and then by external influence acceptance, which had a much weaker link. Whereas our study highlights authentic life as the pivotal component of authenticity, in the study by de Carvalho et al. (2015) that component is self-alienation; the same result is found in studies by Barrett-Lennard (1998) and Van den Bosch and Taris (2014a), who conclude that consistency between individual awareness and an individual’s primary experience (namely, self-alienation) constitutes the fundamental element of being authentic.

There are several motives that could lead us to justify that self-alienation occupies a core position in the authenticity construct, as proposed by Barrett-Lennard (1998), Van den Bosch and Taris (2014a), and de Carvalho et al. (2015). Firstly, it is more likely to find self-alienation among the members of lucrative organizations, such as the ones assessed by the above mentioned authors. In such organizations, it is more probable to identify the features representative of alienation posited by Seeman (1959), such as powerlessness, meaninglessness, normlessness, isolation, or self-estrangement. The members of these organizations adhere to them, in the majority of occasions, as a purely commercial matter, and they remain, endure and face alienating situations because there are not better alternatives in the market. By contrast, research has shown that employees of faith-based organizations are strongly motivated by religious belief (Goldsmith, 2006). This religious motivation leads to a vocational feeling that hinders the development of alienation. Secondly and after all, if between some members of religious organizations emerge feelings of alienation, the natural consequence would be the exit. Regarding lay people, because the market tends to offer better working conditions than faith based organizations. With regard to religious members, because there is not a single instrumental motive that retains them in case of alienation. On the contrary, the order under study in this research is atypical with this regard, since religious members fail to profess perpetual vows, but have to renew them every year if they wish so. Hence, opportunities for abandonment in the event of self-alienation are more likely to occur. Lastly, in this organization there are plenty of family or affective linkages between religious and lay members (nephews, members of the community, and former users of their services that have helped providing an alternative through decent employment…). Such a network of emotional relationships surely stifles the development of the self-alienation between members of the community.

In another sense, the fact that authentic life occupies a core position in the context of a faith-driven organization could be motivated by the existence of a well-defined and rich ideology within this type of organization (with more than three centuries of history in our case), a charisma that is shared and disseminated throughout the entire organization, and a gift that the religious members aspire to share and that extends to the rest of members. Following the path of Jesus, they proclaim the Gospel in serving those who are poor, in other words, God awaits them in those who suffer. To support this goal, the institution combines informal socialization processes with more formal actions oriented to training and mentoring in the Order’s own charisma. In some circumstances, the atmosphere might result in oppression to the extent that particular lay members (school teachers, healthcare professionals within the residences for elderly people, cooks in the social dining rooms, and others) assume – based on reality or imagination – that their survival within the institution would be threatened if they showed themselves as they truly are. As a means of survival, or perhaps because promotion possibilities are different depending on the degree of identification with the institutional ideology, some lay members might feel tempted to hide their feelings or, in other words, behave in an inauthentic manner. In this setting, being authentic perhaps constitutes a poor strategy, as it could lead to rejection by the rest of members (Mengers, 2014).

The fact that the ability to be authentic varies according to the group assessed (which then leads to happiness enhancement, the balance of positive–negative feelings and flourishing) is manifested in Models 2B (religious members), 2C (lay managers), and 2D (lay employees) presented above. Model 2B indicates that for religious members’ wellbeing, there is a single component of authenticity that exerts a significant impact: authentic life (t-statistic: 5.951). However, neither self-alienation (*t*-value: 0.157) nor external influence acceptance (t-statistic: 0.774) show statistically significant results. This means that to feel well, religious members ought to live in a manner that is consistent with their own values and beliefs, which may be easier to achieve given their condition as the “owners” of the Order, enabling them to develop an authentic and fully realized life at every moment.

Additionally, and although it is not a statistically significant result, it is certainly curious that the relationship between external influence acceptance and subjective wellbeing presents a negative sign (path coefficient: -0.128), which suggests a result that differs from the one obtained in the general model. This would mean that among the religious members, external influences would induce different behavior than that which the literature considers to be typical (i.e., Schmid, 2005), since being highly influenced by others would enhance the religious members’ individual well-being. The only study we found that is consistent with this result is that of Van den Bosch and Taris (2014a). These authors found that, in contrast to what they expected, external influence acceptance correlated positively with certain measures of wellbeing such as work satisfaction or the dedication dimension of “work engagement.” The explanation for this anomalous behavior in the case of the religious members might be related to the vocation to serve that permeates the doctrine of the Order. Within the religious members’ charisma, the vocation to serve the poorest human beings is implicit; hence, adjusting and complying with others’ expectations (namely, accepting external influences) not only generates negative effects but also could be considered as acting against the Order’s inspirational principles. Moreover, behaving in accordance with what others desire and complying with their demands and expectations might elicit their gratitude, which might in turn lead to the enhancement of religious members’ subjective wellbeing.

Besides, the majority of the existing theoretical views concerning work-related and occupational issues yield on a vision of individuals who are rather autonomous and that deliberately pursue to make visible what are their objectives, interests, values, and skills at the workplace. In this vein, Blustein (2011) proposes a considerable shift in the understanding of work, which depicts vocational behavior as an intrinsically relational act. This conceptualization of work as a relational act entails that every choice, experience, and link with the work reality is assumed, predisposed, and formed by relationships. The set of advantages and positive features regarding career choice and career development opportunities are strictly constrained to those individuals who experience a certain degree of freedom in their choices – both personal and professional. However, this is not the case for many people, for whom self-determined elections concerning the direction of one’s professional life are not that likely (Blustein, 2006; Savickas et al., 2009).

Duffy et al. (2011) define the term work-calling –namely the personal calling to a particular area of work– to refer to those individuals that perceive it as coming from a superior or “beyond the self” force (i.e., God, a social need, a family legacy). This personal attraction to work comprises the feeling that work might be helpful to people or the broader society, even if it is through an indirect effect. This conceptualization also suggests that a calling enables the attainment of a broader sense of purpose in life. These authors conceptualize work-calling as an ongoing process instead of considering it a singular or isolated event. Hence, rather than applying as a binary concept –you experience it or not at all–, work-calling applies to individuals as a matter of degree. Our results are in line with the previous work of Duffy and Dik (2013), that states that fostering a sense of calling and engaging at the work-place may be an important course to enrich individuals’ well-being.

Model 2C, which focuses on the lay employees, differs somewhat from the religious members’ model. Authentic life remains the main determinant of the model (t-statistic: 12.079); however, in this case, its influence on subjective wellbeing is complemented by the influence of self-alienation (t-statistic: 2.917). Thus, a person’s wellbeing may be principally a mixture of an authentic life and the absence of self-alienation. Similarly, as in the case of the religious members, allowing external opinions to influence oneself is not a significant element of the lay employees’ wellbeing. However, there are two differences between the groups in this respect: first, the non-significance in this case is a very low margin (t-statistic: 1.335), and second, the sign of the relationship is now negative (path coefficient: -0.051), which is more consistent with what the literature has found in this respect. This circumstance might suggest that the service vocation toward others that permeates the religious members’ charisma is perhaps less developed among the lay members who occupy operative jobs within the more than eighty programs that the Order organizes in southern Spain and the Canary Islands.

Finally, Model 2D represents the behavior of lay managers. The results obtained indicate that this group’s wellbeing is exclusively determined by its members’ capacity to be authentic under different circumstances and to be faithful to themselves on most occasions (t-statistic: 3.769). Again, authentic life emerges as the fundamental pillar of the authenticity construct, given that neither self-alienation (t-statistic: 0.374) nor external influence acceptance (t-statistic: 0.993) determine the level of subjective wellbeing of the lay managers. It is paradigmatic that the results obtained for this model are quite similar to those found for the religious members’ model, in terms of both the central and exclusive influence exerted by authentic life and the negative relationship between external influence acceptance and wellbeing (path coefficient: -0.304); thus, the more receptive individuals are to such influences, the greater subjective wellbeing they may attain. This circumstance leads us to presume that the group of lay managers has internalized feelings and ways of acting that are inherent to those of the religious members of the Order, perhaps because assuming managerial positions implicitly leads to the development of a service vocation similar to that of the religious members while serving to the poorest and most vulnerable members of society. In this sense, Sosik et al. (2011) note the existence of a high degree of pressure on lay members who assume managerial positions within religious organizations because they feel compelled to satisfy everyone. This feature, which presents homogeneously among the religious members and lay managers, induces us to argue that the latter are well trained in the Order’s charisma once they occupy a managerial position or they are selected and promoted according to their level of identification and fit with the institution’s ideology. This issue may constitute a research line to develop in future studies.

In conclusion, this study covers two noteworthy research gaps. On the one hand, it provides evidence of the relationship between authenticity and subjective wellbeing within the specific and rarely explored context of faith-driven organizations. On the other hand, it suggests that this relationship is not homogeneous but that it differs among the distinct groups that shape the organization. Our results suggest that within the tridimensional model of authenticity centered on the person, authentic life stands as the central element and bond of the three groups under study; they simultaneously reveal similar behavior between the religious members and the lay individuals who occupy managerial positions, probably because a dedication serving to the poor permeates both groups.

## Implications and Limitations

The concept of authenticity has received scarce attention in the business literature until very recently (Liedtka, 2008). Hence, this research involves some relevant implications, both for theory and for practice. The most important theoretical implications are linked to the role authenticity plays in predicting subjective wellbeing in the context of religious organizations. Understanding how authenticity operates in these organizations might contribute to generating healthier work environments, with members who are more self-aware and who act consistently with their values instead of primarily complying with societal and contextual pressures. In addition, our results are also consistent with the relational approach of leadership proposed by Maak and Pless (2006). Following Freeman and Auster (2011), authenticity is not uniquely shaped by one’s personal values, but by the convergence of our personal values, our background, our network of connections with others, and our own aspirations, which comprise not only individual but also organizational or community goals.

Similarly, from a practical point of view, the results of this study could serve to identify those members within religious organizations who exhibit poor levels of authenticity, in which case preventive policies should be designed to increase individual wellbeing, both in its hedonic and eudemonic forms. Any progress in the management of emotions at work may contribute to the improved functioning of these organizations and may promote enhanced services to aid the community, which is ultimately the reason for the existence of these organizations. Our results also suggest the need to design policies for mentoring and training lay members and collaborators to help them share in and embrace the Order’s charisma. This charismatic training does not have to be carried out in classrooms; instead, it should be approached through coaching and mentoring mechanisms that may enable leaders to guide other members by example. Finally, it should be highlighted that the mimetic connection that seems to exist between the religious members and lay managers represents a source of hope for the future of religious organizations given the current context of vocational scarcity that complicates the maintenance of charismatic faith-driven organizations with lower numbers of religious members.

Nevertheless, this study is not without some methodological limitations, and its results should be interpreted cautiously. Firstly, it should be noted that all the data were obtained through self-reports, which might lead to the existence of common method bias in contrast to the use of objective measures (de Carvalho et al., 2015). The problem of social desirability is another issue that is significant in the study of authenticity and subjective wellbeing, particularly within the type of organization studied in this paper. Moreover, the sample is composed of a single organization, which, although it has an international focus, is located in a specific geographic area (Spain). Therefore, we should be very cautious when attempting to generalize these results to different contexts. Finally, given the transversal design of this study, we cannot affirm the existence of a causal relationship between authenticity and subjective wellbeing.

## Conclusion

Although the PLS methodology is suitable for studies conducted within the social sciences research field present also cautions (e.g., McIntosh et al., 2014) that need to be considered in the results interpretation.

## Author Contributions

All authors listed have made a substantial, direct and intellectual contribution to the work, and approved it for publication.

## Conflict of Interest Statement

The authors declare that the research was conducted in the absence of any commercial or financial relationships that could be construed as a potential conflict of interest.
